# Filtering Biomechanical Signals in Movement Analysis

**DOI:** 10.3390/s21134580

**Published:** 2021-07-04

**Authors:** Francesco Crenna, Giovanni Battista Rossi, Marta Berardengo

**Affiliations:** Measurement and Biomechanics Laboratory, Department of Mechanical, Energy, Management and Transportation Engineering, University of Genova, Via Opera Pia 15A, 16145 Genova, Italy; g.b.rossi@unige.it (G.B.R.); marta.berardengo@unige.it (M.B.)

**Keywords:** dynamic biomechanical measurements, biomechanical dynamic signal filtering, measurement of human movement, biomechanics, kinematic analysis

## Abstract

Biomechanical analysis of human movement is based on dynamic measurements of reference points on the subject’s body and orientation measurements of body segments. Collected data include positions’ measurement, in a three-dimensional space. Signal enhancement by proper filtering is often recommended. Velocity and acceleration signal must be obtained from position/angular measurement records, needing numerical processing effort. In this paper, we propose a comparative filtering method study procedure, based on measurement uncertainty related parameters’ set, based upon simulated and experimental signals. The final aim is to propose guidelines to optimize dynamic biomechanical measurement, considering the measurement uncertainty contribution due to the processing method. Performance of the considered methods are examined and compared with an analytical signal, considering both stationary and transient conditions. Finally, four experimental test cases are evaluated at best filtering conditions for measurement uncertainty contributions.

## 1. Introduction

The biomechanical study of human movement requires a strict integration between experimental data and models to describe motion patterns [1,2]. Moreover, when dealing with physical parameters that cannot be directly measured, a model-based inverse-dynamics problem has to be solved, which requires the measurement of kinematic quantities, including position, velocity and acceleration of reference points, as well as angular displacement and relative derivatives of body limbs.

State-of-the-art measurement systems for kinematic analysis in biomechanics include video or inertial sensors. In the first scenario, a preliminary calibrated video system is used to measure the position in the two or three-dimensional space, according to gesture’s space development (2D measures can fit sufficiently some gestures, while others are intrinsically 3D). The video shows a set of reference markers on the subject corresponding to very evident dots with respect to the background. Experimental signals resulting from the measurements are positions. The second scenario is composed of an inertial measurement unit (IMU), consisting of accelerometers, gyroscopes and magnetometers, placed on the body segment, measuring its orientation in the space. Experimental signals resulting from the measurements are angles. In both scenarios, some noise affects the measurements, mainly due to electronics and processing of the IMU signals, or to illumination, fast movements, camera resolution and focus in the video scenario [3].

To obtain velocities and accelerations from position and angle measurements, a differentiation process and low pass filtering is necessary. Filter selection and setup are critical because noise might affect numerical derivatives [4,5].

For this reason, differentiation procedures and their characterization has been analyzed from several points of view in literature. Regarding specific biomechanics’ application, three frequently used approaches to differentiation can be identified: (1) numerical differentiation followed by low pass filtering, (2) polynomial local approximation and direct differentiation, and (3) optimal Fourier filtering.

As regards the first Approach, several papers available in literature investigating low pass filtering performances [6] by comparing filtering results to standard gait patterns, with and without added noise, or introducing simulated gait patterns as reference [7,8,9]. The effectiveness of such studies is limited by the need to adapt filters’ setup to the specific signal and to the considered derivative order.

The consistency of the polynomial approach with the model and its degrees of freedom is considered by [10,11,12] addressing the effect of differentiation methods on modelling, and on its use in inverse dynamics analysis results. Numerical differentiation methods are compared to an experimental reference signal from a worn accelerometer in [13] introducing an experimental reference signal.

The Fourier approach bases on biomechanical signal spectral content to identify a convenient filter bandwidth. Such approach presents a good performance with the burden of heavier computation. An interesting compromise is discussed in [14,15,16,17] optimizing the spectral reconstruction of the biomechanical signal for successive analytical differentiation.

Polynomial interpolation was increasingly adopted in biomechanics following Savitzky and Golay’s computational efficient procedure [1,18,19]. Such an approach presents the advantage to obtaining the analytical differentiation, point by point, considering the interpolating polynomial. On the other hand, the polynomial filter set up is not straight forward due to nonlinear behavior [20].

It is worth noting that the majority of the papers consider the differentiation problem applied to gait one of the most studied human gesture in biomechanics. Interesting different developments in biomechanical studies include a variety of upper limbs sports gestures including ergonomic studies in human–machine interaction [21] or repeated movements in working activities [22]. A general approach addressing measurement uncertainty contribution due to the differentiation process seems to be appropriate.

Most literature considers rms errors between reference and numerically differentiated signals as differentiation/filtering performance indicators. In order to fulfill constraints of different biomechanical applications, specific performance indicators are required. Rms error, the most common in literature, is a good general indicator applied to the analysis or modelling of an overall, rather slow, gesture such as gait. In sports acceleration, peaks might be essential to characterize or optimize gesture performance, and their values heavily influence the measurement of articular forces and moments through an inverse dynamic model.

The energetic analysis is another aspect to consider, since it can be influenced by differentiation errors. When only short acquisitions are available, border effects might be dominant. Such effects, which are of great importance when using numerical filtering, are rarely analyzed [23,24,25]. Except for Fourier methods, differentiation by filtering is based on a processing window in which borders can show an abnormal behavior, due to the filter action. This may be eliminated when long sequences of a repetitive (periodic) gesture are available, but it highly impacts those cases in which a record of a single gesture is available. In such cases, specific performance indicators are needed to characterize the differentiation/filtering procedure, and a rough indication of the possible differentiation error is indispensable when comparing results from different experiments, or a result from a trial with a normality range.

The purpose of this work is to propose a possible evaluation scheme, to better understand filter performance according to the above limits discussed regarding the existing proposed methods. We consider both signal and its derivatives, by using an analytic reference signal and a set of experimental test cases [26]. The use of an analytical reference signal makes possible the evaluation of measurement uncertainty, giving useful indications on the reliability of the obtained values. Tests cases will cover specific aspects illustrated above.

The set of selected filtering methods will be discussed, critically analyzing their main features. A reference signal, with added random noise, is adopted, where derivatives are analytically computed to become the reference. Performance evaluation will consider both uncertainty on the filtered signal, in terms of signal to noise ratio, and transient performance on signal and its derivatives.

Some practical indications for selecting and defining the most convenient parameters of the filter are supplied when discussing performance criticalities of analyzed cases. As a last step, a few experimental test cases are described as samples of possible application.

## 2. Materials and Methods

Low pass filtering in biomechanics can be used in both kinematic and kinetic experimental data. In the former case, as already depicted, we can identify two main goals signal to noise ratio improvement and differentiation. In the latter, the main advantage is to reduce noise contribution in force measurements, in order to use such information in inverse kinetics procedures.

In both cases, common cut off frequencies are in the range between 3 and 10 Hz [1] with values around 6 Hz, such as in the biomechanical simulator OpenSim [27,28] default parameters.

In this paper, the cut off frequency is 10 Hz which represents all the considered experimental test cases’ bandwidth and most biomechanics’ applications range.

### 2.1. Differentiation and Filtering Methods

Numerical differentiation in biomechanics is usually carried out according to the method proposed by Winter and available in [1]. Winter method considers, for each sample, a mean of the finite differences with both previous and following samples. A low pass filtering is added to reduce noise effects. In some cases, before differentiation, a low pass filtering is preliminary applied to improve signal to noise ratio (SNR). Several low pass filtering methods are available in literature, in the following we will focus on:

- Moving average (MA) filter: it is a window-based filter which is a good introduction for polynomial filters;
- Butterworth zero phase (BZP) low pass filter: one of the most used in biomechanics, proposed by Winter in [1] and analyzed in [29];
- Savitsky–Golay polynomial filters [18]: very commonly used in biomechanics.

Let us the briefly introduce and discuss these three methods.

#### 2.1.1. Moving Average (MA) Filters

Although its frequency response is not as performing as for other filter types, this filtering method is amply used thanks to its intuitive behavior. It is based on an average window, whose time duration determines its bandwidth, and the consequent number of points to be averaged is determined by the sampling frequency. When short windows are involved, together with slow sampling rates, the limitation associated with the minimum number of three points, becomes critical.

The window is usually based on an odd number of samples: 2M + 1, with M positive integer, causing a transient behavior when the filter is applied to signal record’s extremities, affecting M samples after start and before the end. Such effects are particularly evident when dealing with derivatives. Special smoothing windows, such as Hanning, might be used to obtain a weighted moving average filter, smoothing transient effects but altering filter bandwidth. In the following we will consider a window length, for the MA filter, with cutoff frequency at 10 Hz.

#### 2.1.2. Linear Filter

Among linear filters, the Butterworth filter is very common in filtering biomechanical signals [1,2,30,31]. After a proper design to obtain the desired cut off frequency, the filtering is generally applied two times, in forward and reverse directions on the signal time history, to obtain a zero-phase filter.

In the following, we will consider a second order 10 Hz cut off Butterworth filter, applied two times to the time series, obtaining a frequency response of fourth order. The Fourth order is typical for biomechanical application as depicted in [1].

As in the previous case, linear filtering presents a transient behavior dependent on filter set up.

#### 2.1.3. Polynomial Filter

Savitzky–Golay, SG, is a polynomial filter, whose parameters are determined by least square procedure, on a window of points centered on the point of interest [30,31,32,33,34,35]. The filter set up requires specification of the polynomial order and of the number of points in the window. This filter is similar to the MA filter, since it uses a zero-th order polynomial, or average, fitted on a moving set of samples as defined by window length. However, SG has a nonlinear behavior, since it operates at higher orders, so bandwidth analysis and filter set up is not trivial. Cut off frequency depends on both order and window length and the same bandwidth can be achieved with different combinations, highly affecting transition band behavior. A SG filter introduction, giving useful frequency cut off and bandwidth indications is available in [20].

**Figure 1:**
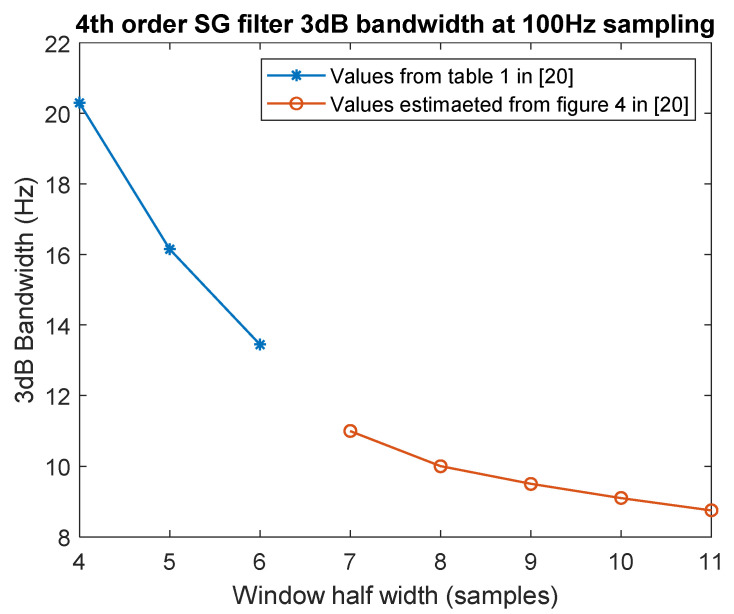
The 3 dB bandwidth for SG applied to a signal sampled at 100 Hz, as a function of window length. Data from [20].

The main advantage of this method, from the point of view of biomechanical motion analysis, is that once polynomial coefficients have been determined in a point, the signal derivatives are obtained by analytical derivation, therefore, avoiding numerical derivative procedures. This advantage often overcomes the difficulties in setting up a proper filter for noise reduction. We have selected a fourth order SG filter, as widely used in biomechanics, and to determine the proper window length we used reference [20]. Form this reference it is possible to identify an empirical relationship between order, window length and cut off frequency. We are interested in obtaining the same MA and BZP cut off frequency, by using a fourth order SG filter, so we are constrained to window length selection. To this purpose we have considered data in [20], using both graphical presentations and tables, to reconstruct the cut off frequency to window length empirical relation depicted in Figure 1.

The curve near 10 Hz is rather flat including different possible lengths, some preliminary tests were necessary to verify filter bandwidth considering amplitude reduction. We have tested windows of 15–17-and 19 samples (7–8 and 9 samples on Figure 1) to identify the 17 points window, as the nearest to the 10 Hz cut off.

### 2.2. Reference Analytical Signals

In order to compare filters performance on signal and its derivatives, we need a reference analytical signal. We propose here to consider a pure harmonic signal, Xa(t), defined considering frequencies involved in some typical biomechanical investigations, such as gait and hopping analysis.

In the latter case, a typical protocol considers the most preferred hopping frequency of 2.2 Hz, which is set by a metronome, to ensure a stable gesture repetition [36,37]. In walking studies, generally the step pace is about 2 Hz. Some harmonics has to be considered in the measurement set up, leading to a frequency band of about 5 ÷ 10 Hz [1].

We have generated time histories of the reference signal and its derivatives from the analytical definition with a sampling frequency of 100 Hz, $Xa\left(t_{i}\right),\;$which is compatible with most biomechanical data acquisition systems. Of course, it is possible to investigate different parameters configuration both for sampling and fundamental frequency.

Some noise is added to the analytical reference to better simulate the experimental situation. This noisy signal $X\left(t_{i}\right)$, sampled at 100 Hz, will be the test signal input for the filters we are considering.

### 2.3. Experimental Test Cases

Once filters are characterized by considering the analytical reference signal and the performance parameters presented in the following Section 2.4, it will be possible to apply the same methods to some experimental test cases which are typical for the biomechanical application we are considering. We will consider kinematic measurements of different gestures:

- Standard gait analysis—one gait cycle only;
- In place hopping according to [36,37];
- Voluntary self-oscillations around an upright stable positions;
- Maximum height jump [38].

### 2.4. Test Procedure

Once the analytical signal has been generated together with its first and second derivatives, at the required sampling frequency, some noise is added to the original signal, before entering the processing phase, as reported in Table 1.

Analytical signal offers a reference for both time signal and its derivatives. After noise addition, it is possible to numerically differentiate the signal, by using, for example, the method recommended by Winter in [1]. This method proceeds point by point along the signal history, considering an average of the numeric differences obtained with previous and successive points.

**Figure 2:**
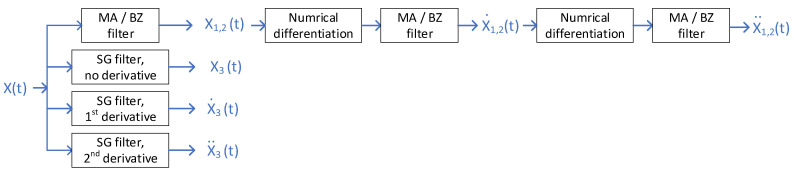
Signal processing to obtain first and second derivative.

Raw results are then filtered according to the procedures depicted in Figure 2:

MA and BZP filters are applied to the signal. Then the filtered signal is differentiated and filter is applied again. First derivative is differentiated again and MA/BZP filters are applied. Hence each differentiation order n is filtered n + 1 times.

Such numerical differentiation and filtering procedures are not required in the SG case, since one of its peculiar advantages is the possibility to obtain derivatives directly from the polynomial coefficients. Table 2 presents filters parameters that we have considered in this study.

Once we have obtained filtered signals and their derivatives, we can evaluate performance indicators by considering their difference with the reference analytical signal.

### 2.5. Performance Indicators

Since we rely on an analytical reference, a performance indicator may be developed starting from the point-by-point difference between signal and its derivatives, obtained after differentiation and filtering procedures, with the corresponding references. This can be considered as an estimation of the measurement error, since the reference simulates the measurand and the signal is the output of the measurement procedure. In order to have an overall synthetic parameter, it is possible to consider the rms error value, calculated on all recorded time history, or all available N samples.

(1) $$E_{rms}=\sqrt{\frac{1}{N}\overset{N}{\underset{i=1}{\sum }}{\left(X\left(t_{i}\right)-Xa\left(t_{i}\right)\right)}^{2}}$$

This gives us an absolute picture of the situation that can be normalized to the analytical rms reference, $Xa_{rms}$, giving a figure of the overall relative error.

(2) $$E_{rel}=100\frac{E_{rms}}{Xa_{rms}}(\%)\;\mathrm{or}\;E_{rel}=20\;log_{10}\left(\frac{E_{rms}}{Xa_{rms}}\right)\;(\mathrm{dB})$$

The same relative approach can be expressed as a signal to noise ratio, SNR, which is perhaps more informative as regards signal processing. Such overall evaluation fails in identifying specific aspects such as transient behavior at time history extremes and the eventual error on signal peaks.

Transient behavior is important when dealing with limited in time signal histories, for example a single gait recording. In such cases, border effects could be higher than in the central part, so a quantification of the border error according to (3) could be useful to optimize the filter and evaluate the uncertainty in these specific parts.

(3) $$E_{win}=\sqrt{\frac{1}{2Win}\left(\overset{Win}{\underset{i=1}{\sum }}{\left(X\left(t_{i}\right)-Xa\left(t_{i}\right)\right)}^{2}+\overset{N}{\underset{i=N\;-\;\mathrm{Win}}{\sum }}{\left(X\left(t_{i}\right)-Xa\left(t_{i}\right)\right)}^{2}\right)}$$

$E_{win}$ considers rms error only in the first and last filtering windows, Win, in the time history recording. The border error is then normalized following the same principle as in (2) to obtain $E_{bor}$.

Peak level evaluations are required in some gestures, commonly in sports investigations, for example acceleration peaks during a jump. In such cases, an overall error evaluation is not sufficient, and we need a specific performance indicator. A certain number $N_{peaks}$ of peaks are identified in the analytical reference absolute value obtaining their positions, $t_{k},\;$and levels, $\left|Xa\left(t_{k}\right)\right|$. Then peak levels are averaged obtaining a reference for the peak value.

(4) $$Peak_{ref}=\frac{1}{N_{peaks}}\overset{N_{peaks}}{\underset{k=1}{\sum }}\left|Xa\left(t_{k}\right)\right|$$

Rms difference between peak levels in the same positions $t_{k}$, for reference and filtered signals is evaluated:

(5) $$RMS_{peak}=\sqrt{\frac{1}{N_{peaks}}\overset{N_{peaks}}{\underset{k=1}{\sum }}{\left(\left|X\left(t_{k}\right)\right|-\left|Xa\left(t_{k}\right)\right|\right)}^{2}}$$

Finally, the ratio between error and reference level gives the dynamic performance indicator in %:

(6) $$E_{peak}=\frac{RMS_{peak}}{Peak_{ref}}$$

Figure 13 presents graphically the zones of the time signal interested by performance indicators.

Now we are going to apply the performance indicators summarized in Table 3, to:

- The signal itself evaluating only the low pass filtering effect, and;
- Its first and second derivatives.

## 3. Results

### 3.1. Signal Filtering

**Figure 3:**
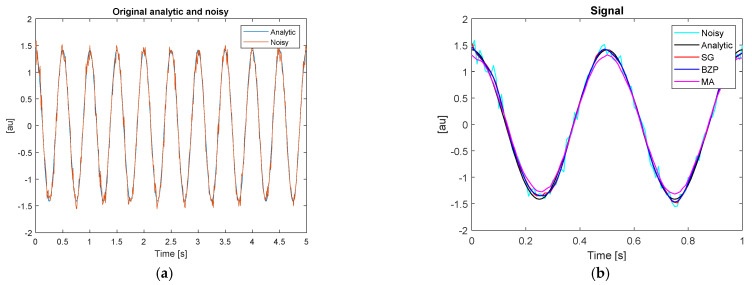
Reference analytical and noisy signals. (**a**) Time histories; (**b**) filter effect detail for polynomial, SG, (red), moving average, MA, (magenta) and Butterworth zero phase, BZP (blue), compared to the reference analytic (black) and noisy (cyan) signals.

Analytic reference and noisy signals are presented in Figure 3a. The filter effect is presented in a detailed view in Figure 3b.

**Figure 4:**
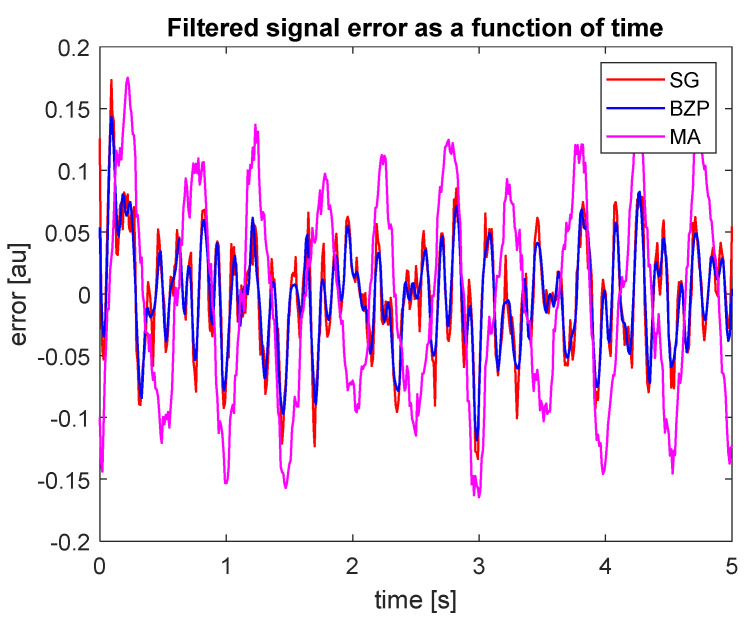
Point by point error as difference between each processed signal and the analytical reference.

The MA filter performance is a bit lower than the others, as expected. It is difficult to identify, from these graphs, a best performance option. Hence, for each signal, we computed a point-by-point error as the difference towards the analytical reference, to evaluate some significant parameters as described in Section 2.5. Figure 4 represents the point-by-point error chart. The border effect is evident on the left for the first about 0.2 s. In Table 4, we present some performance figures.

### 3.2. First Derivative

**Figure 5:**
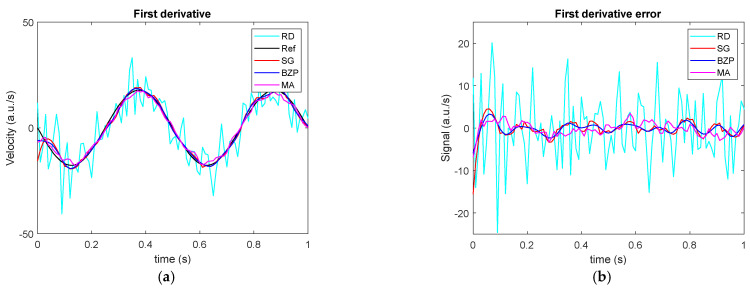
Signal differentiation. (**a**) Time histories for analytical signal first derivative (Reference, Ref), numerically differentiated signal (raw differentiation, RD), differentiated and filtered by polynomial (SG), Butterworth (BZP) or moving average (MA), and; (**b**) first derivative errors for all the methods.

We now take into consideration the derivatives. Figure 5 and Table 5 show that, while in time filter performance can be intuitively estimated considering bandwidth or filter nonlinear properties, after the differentiation process, filter behavior is much less predictable.

### 3.3. Second Derivative

**Figure 6:**
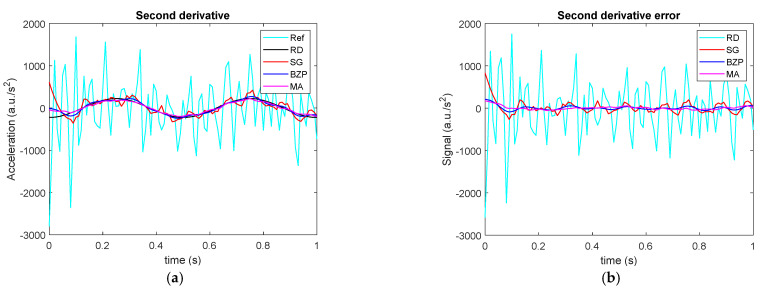
Signal double differentiation. (**a**) Time histories for analytical signal second derivative (Reference, Ref), numerically differentiated signal (raw differentiation, RD), differentiated and filtered by polynomial (SG), Butterworth (BZP) or moving average (MA), and (**b**) the second derivative errors for all the methods.

Moving now to the second derivative, errors are much more evident as shown in Figure 6 and Table 6.

### 3.4. Experimental Test Cases

In this section, we apply characterized methods to a set of experimental signals useful in the biomechanical analysis of the gestures presented in Section 2.3.

#### 3.4.1. In Place Hopping

**Figure 7:**
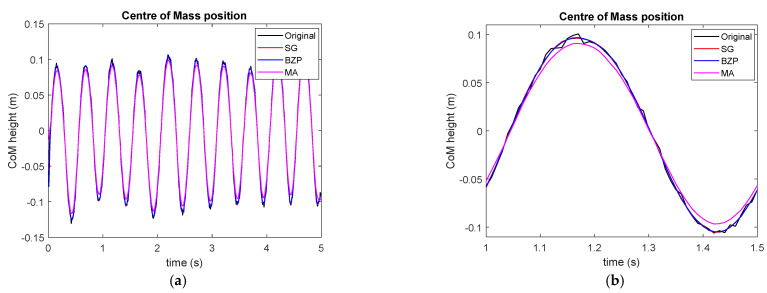
CoM vertical position during hopping. Complete acquisition (**a**) and detail (**b**).

We start considering the vertical movement of a point approximately near the center of mass of the subject during repeated hopping at a regular pace. The signal is approximately periodical, as presented in Figure 7, and acquisition lasts for several repetitions, so we can manage border effects by cutting the central part of the signal after filtering.

**Figure 8:**
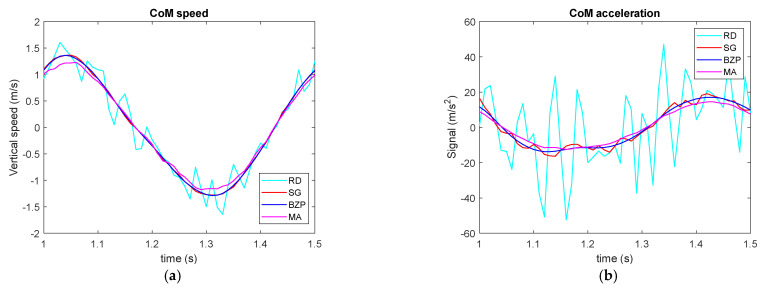
CoM vertical speed (**a**) and acceleration (**b**).

Figure 8 presents signal derivatives or CoM vertical speed and acceleration. Raw differentiation (in cyan) as expected creates a very large noise. While BZP (in blue) presents a smoother behavior compared to SG (in red). MA (in magenta) underestimates signal peaks and it seems to have an insufficient bandwidth, even if the three filters are designed for the same cut off frequency.

**Figure 9:**
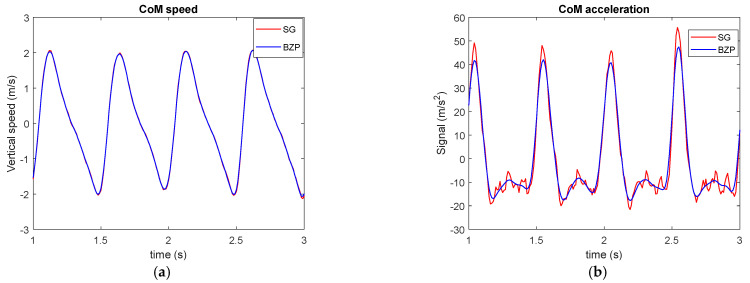
CoM vertical speed (**a**) and acceleration (**b**) in forced hopping.

Figure 9 presents first and second derivatives for the same gesture but requiring the subject to force the hopping while maintain the indicated rhythm. In this case, we limited comparison to SG and BZP since they demonstrated to be best choices when interested in differentiation.

The performance here is equivalent in the first derivative while in the second derivative SG seems more noise sensitive and captures larger acceleration peaks, while the smother BZP enables the identification of the flight period in which acceleration is near gravity.

#### 3.4.2. Gait

**Figure 10:**
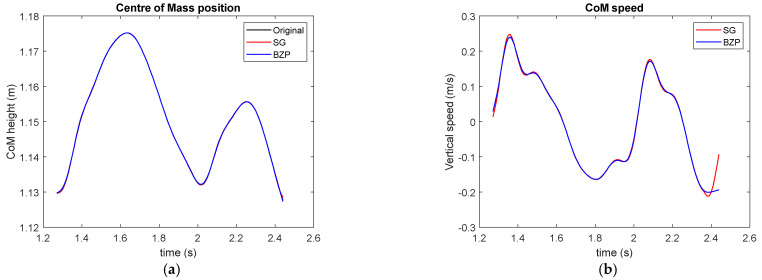

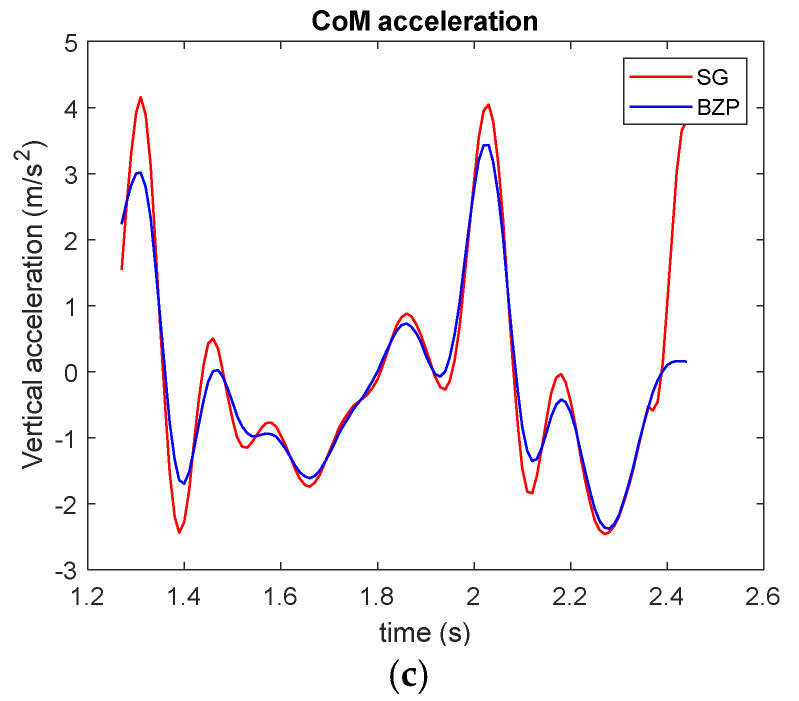
CoM vertical position (**a**), speed (**b**) and acceleration (**c**) during a gait cycle.

In gait analysis, available signals often refer to a single gait cycle. This is particularly critical due to the border effects. Figure 10 presents CoM height during a cycle and its vertical speed and acceleration. Border effects are evident when considering speed and acceleration. Near borders SG-BZP performance parameters show 14%–28% errors respectively for the first derivative and 170%–59% for the second derivative. This is evident in the acceleration graph when considering the right border, where SG and BZP signals abruptly deviate.

#### 3.4.3. Self-Oscillations

**Figure 11:**
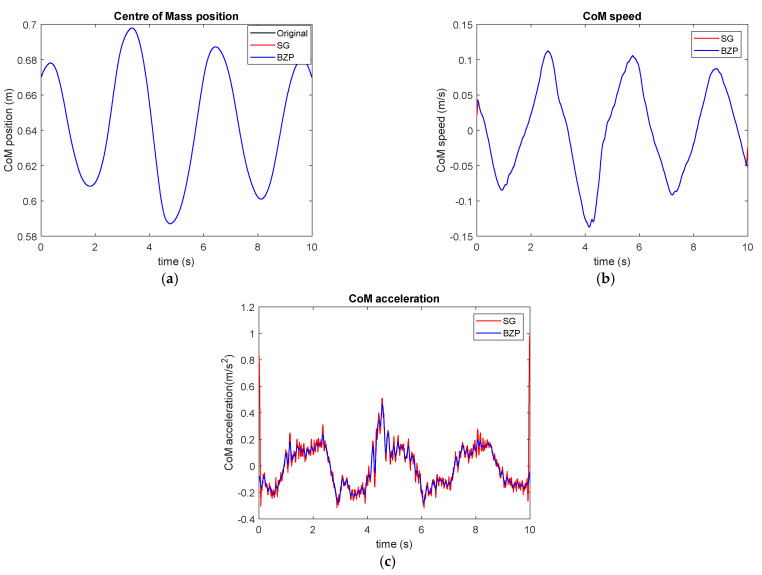
CoM horizontal position (**a**), speed (**b**) and acceleration (**c**) during voluntary oscillation around a stable and constant position.

In this gesture, the horizontal position of the CoM of a subject who voluntary oscillates back and forward, having care to maintain feet stable and still on the ground and legs as rigid as possible. This signal is not exactly periodical, as presented in Figure 11, since the subject is not required to maintain a fixed rhythm; the periodicity is due to the movement back and forward. Possible oscillations in these specific conditions are very small, hence measurement results are generally subject to a large amount of noise as compared with the movement amplitude.

The noise effect is more evident, in particular for the acceleration chart. BZP remains smoother than SG and presents lower border effects.

#### 3.4.4. Maximum Height Jump

**Figure 12:**
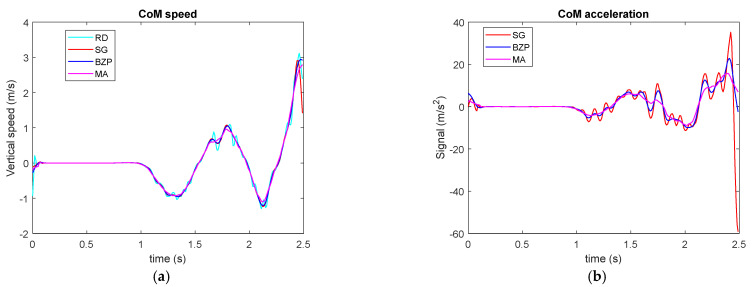
CoM vertical speed (**a**) and acceleration (**b**) in a maximum height jump.

The maximum height jump is commonly used to evaluate athletes’ explosive performance. Experiments include kinematics and/or kinetic measurements through force platforms. This signal is not periodic, since the subject intention is to produce a force pulse on ground to obtain maximum vertical acceleration and reach maximum vertical height. We consider CoM vertical position measurements until the subject’s take off. Figure 12a presents CoM speed showing pre jump movements and a peak value before taking off. From the speed signal, we computed acceleration as presented in Figure 12b, where we have excluded the raw computation since, due to noise, it would saturate the graph scale. The difficulty in peak value measurement is evident: discrepancies between the three methods are rather large.

Considering $E_{peak}$, defined in Equations (5) and (6), as a standard deviation we can introduce a peak acceleration uncertainty due to computation only, calculated as a 2$\;E_{peak}$ error, obtaining the values in Table 7, in which large intervals confirm the graphical impression, but they overlap for all three methods, supporting the validity of the proposed approach.

## 4. Discussion

**Figure 13:**
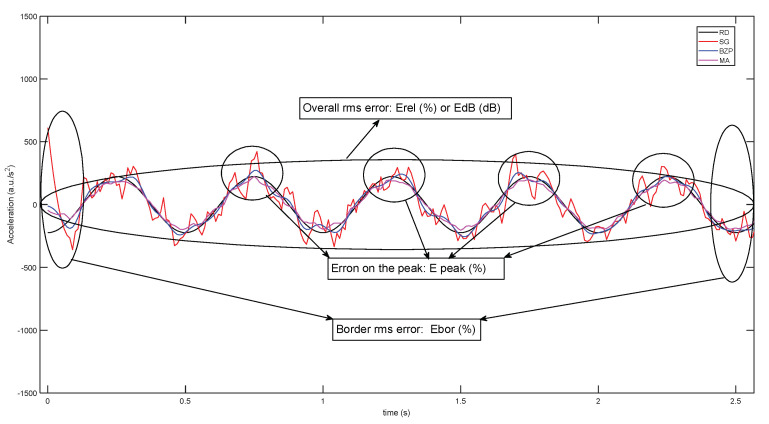
Error parameters to evaluate differentiation/filtering procedures and their evaluation zones on signal time history.

The introduction of a reference signal and its analytical derivatives enables a quantitative evaluation of numerical differentiation procedures. For this purpose, we considered a set of error parameters for specific aspects of the signal and its derivatives: overall error, error in recording borders, error on the peaks, as presented in Figure 13. A parameter can be selected according to the objectives of the biomechanical analysis that is carried out and consequently an estimation of the possible error due to the differentiation/filtering procedure is possible. For example, a general analysis of a periodic gesture may require a general error evaluation and some indications of possible border effects. If the acquisition is rather short and the gesture starts very near recording border the border error can give useful information. If the interest is on peaks, (for example, acceleration peaks in sports gestures), the peak error is the most suitable parameter.

Even if in typical biomechanical applications, kinematic signal processing is off-line and does not require particularly fast computation, each method requires a suitable and efficient implementation, however, using the standard algorithms available for example in Matlab^®^, the computation time is limited to few milliseconds for a 2.5 s signal and its derivatives, for all of the methods.

When evaluating measurement uncertainty, the instrumental effects are significant [3] but when dealing with biomechanical models, other contributions should be considered [39] and, among them, the differentiation and filtering procedure might be important. The proposed approach gives an estimation of possible errors according to the selected filtering procedure.

An evaluation of the errors for the simulated signals at 2 and 5 Hz and the three differentiation orders considered, shows that:

- When a time signal filtering is considered Butterworth and polynomial filters have almost the same performance in the three areas, moving average is acceptable, even if slightly less efficient;
- When considering derivatives, the moving average filter is less performing than the others, but its performance is almost unvaried between 2 and 5 Hz and for differentiation order, hence this approach shows to be simple and robust;
- In first and second differentiation, Butterworth has a more stable behavior with respect to frequency and in general, it shows a better performance in the borders if compared with polynomial methods. Performance on peak level measurement is acceptable and robust.
- The Savitsky–Golay filter seems to improve its performance in first and second derivatives, by increasing the reference frequency from 2 to 5 Hz. The differences in performances create some difficulties for uncertainty evaluation when the frequency content of the signal is not “a priori” known.

It is worth noting that in this study, a similar set up for all the filters was considered. The different performance of the Savitsky–Golay polynomial filter for 2 and 5 Hz, might indicate that a specific tuning is required. Such a tuning is not simple to design a priori, it would require a strict frequency band of interest and might require several attempts. On the other hand, Butterworth filtering is much simpler to tune, moreover, it is robust enough to perform in an acceptable way for all the differentiation orders for the considered frequencies, and in all areas of interest. According to the proposed evaluation frame, it seems the best choice. Our evaluation limits are due, for example, to the pure harmonic reference signal. A further step might include simulation of more complex signals maintaining the possibility to have a reference analytical derivative. As regards the filter setup, we can suppose that a filter specific tuning, might improve performance, but it would change the evaluation frame that in this paper considers the same order and cutoff frequency for all the filters. While, as in regards to filter type, the proposed approach can be used to evaluate other differentiation and filtering procedures or application of pre-treatments, such as the Hanning smoothing windows in the moving average filter, to limit border effects.

In general, this work defines a basic evaluation procedure and supplies the biomechanical experimenter some useful indication, to evaluate uncertainty contributions due to differentiation and filtering of biomechanical signals.

## 5. Conclusions

The three low pass filtering procedures—moving average, Butterworth, and polynomial, applied to differentiation of biomechanical signals have been studied considering both as a simulated reference and experimental kinematic signals.

Beside the rms error, we have introduced other performance parameters focusing on error on the peak values, as well as border errors.

In order to compare filter performance, a standard set up for all the methods was considered, as illustrated in Table 2. With such constrains, it emerges that the Butterworth zero phase low pass filter presents the most robust performance in all differentiation conditions. Savitsky–Golay polynomial filtering is valid, but it presents different performances at different frequencies. To improve its performance, it requires fine tuning depending on both derivative order, signal type and frequency, and the objective of the analysis.

Moreover, the reference signal has enabled a quantification of the differentiation contribution to the measurement uncertainty for a differentiated signal such as velocity or acceleration. Uncertainty contribution are defined according to the considered parameters, for the signal in general, for the signal’s borders, or for peak value evaluation.

Further experimentation will be based on more complex simulated signals considering different filters setups.

## Figures and Tables

**Table 1:** Reference analytical signal parameters.

Analytical Signal Parameters	
Sampling frequency $f_{s}$	100 Hz
Analytical frequency $f_{0}$	2–5 Hz
Analytical amplitude $Xa_{0}$	$\sqrt{2}$ (a.u.)
Analytical rms value $Xa_{rms}$	1 (a.u.)
Random noise rms	0.1 (a.u.)
X SNR	20 dB

**Table 2:** Low pass filters characteristics.

Filter	Parameters
Moving average, MA	Window length 5 samples–$f_{cut}≅10\;\mathrm{Hz}$
Butterworth zero phase filter, BZP	Second order two passages → 4th order–$\;f_{cut}=10\;\mathrm{Hz}$
Savitsky–Golay polynomial filter, SG	Fourth order window length 17 samples–$f_{cut}≅10\;\mathrm{Hz}$

**Table 3:** Performance indicators summary.

Indicator	Description	Focus
$E_{rel}$ (%)	Error rms on reference rms in percentage	Overall performance
$E_{dB}$ (dB)	Error rms on reference rms in dB	Overall performance
$E_{bor}$ (%)	Border error rms on reference rms in percentage	Border performance
$E_{peak}$ (%)	Error on peak evaluation on reference peak value in percentage	Impulsive performance

**Table 4:** Performance indicators for low pass filtering of noisy 2 and 5 Hz time signals.

Signal/Indicator	$E_{rel}\;(\%)$		$E_{dB}\;(\mathrm{dB})$		$E_{bor}\;(\%)$		$E_{peak}\;(\%)$	
Noisy	10	10	20 ^1^	20 ^1^	14	13	4.7	7
MA filtered	4.7	10	27	20	6.5	14	3.4	10
BZP filtered	4.0	6.6	28	24	6.3	11	2.6	6
SG filtered	4.6	4.6	27	26	7.3	7	2.9	3

^1^ Imposed by simulation and confirmed by performance evaluation.

**Table 5:** Performance indicators for the first derivative obtained by raw numerical differentiation (RD) and low pass filtering or by polynomial filtering (SG) on the original 2 and 5 Hz noisy time signals.

Signal/Indicator	$E_{rel}\;(\%)$		$E_{dB}\;(\mathrm{dB})$		$E_{bor}\;(\%)$		$E_{peak}\;(\%)$	
Raw differentiation—RD	55	22	5	13	69	28	48	16
RD + MA filtering	16	13	16	18	19	20	12	12
RD + BZP filtering	8.6	13	21	17	14	25	5.4	12
SG filtering	13	9	18	21	28	11	6.4	8

**Table 6:** Performance indicators for the second derivative obtained by raw numerical differentiation (RD) and low pass filtering or by polynomial filtering (SG) on the original 2 and 5 Hz noisy time signals.

Signal/Indicator	$E_{rel}\;(\%)$		$E_{dB}\;(\mathrm{dB})$		$E_{bor}\;(\%)$		$E_{peak}\;(\%)$	
Raw differentiation–RD	374	59	−11	5	630	88	202	43
RD + MA filtering	39	23	8	13	65	42	28	21
RD + BZP filtering	23	21	13	13	59	50	14	18
SG filtering	64	12	4	19	170	29	32	7

**Table 7:** Peak acceleration values for the maximum height jump including a ± 2σ uncertainty due to differentiation/filtering methods, evaluated according to reference signal tests in Table 6.

Method	Peak Acceleration (m/s^2^)
RD + MA filtering	16 ± 8
RD + BZP filtering	23 ± 7
SG filtering	35 ± 14

## Data Availability

The data presented in this study are available on request from the corresponding author. The data are not publicly available due to privacy reasons.
